# Formation and Expansion of Memory B Cells against Coronavirus in Acutely Infected COVID-19 Individuals

**DOI:** 10.3390/pathogens11020186

**Published:** 2022-01-29

**Authors:** A. Karim Embong, Phuong Nguyen-Contant, Jiong Wang, Preshetha Kanagaiah, Francisco A. Chaves, Theresa F. Fitzgerald, Qian Zhou, Gabrielle Kosoy, Angela R. Branche, Benjamin L. Miller, Martin S. Zand, Mark Y. Sangster, David J. Topham

**Affiliations:** 1 David H. Smith Center for Vaccine Biology and Immunology, Department of Microbiology and Immunology, University of Rochester Medical Center, Rochester, NY 14620, USA; AbdulKarim_Embong@urmc.rochester.edu (A.K.E.); Preshetha_Kanagaiah@urmc.rochester.edu (P.K.); Francisco_Chaves@urmc.rochester.edu (F.A.C.); Theresa_Fitzgerald@urmc.rochester.edu (T.F.F.); Mark_Sangster@urmc.rochester.edu (M.Y.S.); 2 ACM Global Laboratories, Rochester, NY 14624, USA; nqthucphuong@gmail.com; 3 Division of Nephrology, Department of Medicine, University of Rochester Medical Center, Rochester, NY 14620, USA; Jiong_Wang@urmc.rochester.edu (J.W.); Qian_Zhou@urmc.rochester.edu (Q.Z.); Martin_Zand@urmc.rochester.edu (M.S.Z.); 4 Department of Biochemistry and Biophysics, University of Rochester, Rochester, NY 14620, USA; Gabrielle_Kosoy@urmc.rochester.edu (G.K.); Benjamin_Miller@urmc.rochester.edu (B.L.M.); 5 Department of Medicine, University of Rochester Medical Center, Rochester, NY 14620, USA; Angela_Branche@urmc.rochester.edu

**Keywords:** SARS-CoV-2, B cells, antibodies, memory

## Abstract

Infection with the β-coronavirus SARS-CoV-2 typically generates strong virus-specific antibody production. Antibody responses against novel features of SARS-CoV-2 proteins require naïve B cell activation, but there is a growing appreciation that conserved regions are recognized by pre-existing memory B cells (MBCs) generated by endemic coronaviruses. The current study investigated the role of pre-existing cross-reactive coronavirus memory in the antibody response to the viral spike (S) and nucleocapsid (N) proteins following SARS-CoV-2 infection. The breadth of reactivity of circulating antibodies, plasmablasts, and MBCs was analyzed. Acutely infected subjects generated strong IgG responses to the S protein, including the novel receptor binding domain, the conserved S2 region, and to the N protein. The response included reactivity to the S of endemic β-coronaviruses and, interestingly, to the N of an endemic α-coronavirus. Both mild and severe infection expanded IgG MBC populations reactive to the S of SARS-CoV-2 and endemic β-coronaviruses. Avidity of S-reactive IgG antibodies and MBCs increased after infection. Overall, findings indicate that the response to the S and N of SARS-CoV-2 involves pre-existing MBC activation and adaptation to novel features of the proteins, along with the potential of imprinting to shape the response to SARS-CoV-2 infection.

## 1. Introduction 

The severe acute respiratory syndrome coronavirus 2 (SARS-CoV-2) is the seventh member of the human coronaviruses. It belongs to the genus β-coronavirus, together with the highly lethal SARS-CoV (2003) and middle east respiratory syndrome (MERS) CoV, as well as the seasonal common cold human coronavirus (HCoV) OC43 and HKU1. The profile of immune response following seasonal coronavirus infection had not received much attention, with recent reports indicating that the immunity against human coronaviruses is either short-lived or the viruses evolve to escape immune pressure [1,2]. Moreover, the rise of recent SARS-CoV-2 variants of concern has also raised questions on the role of serum Abs to provide long-lasting immunity [3,4,5]. Serological studies have shown that SARS-CoV-2 infection generates a strong circulating Abs response against the surface spike (S) glycoprotein and the internal nucleocapsid (N) protein [6,7,8,9]. Infection also generates serum Abs response against other viral proteins, including the non-structural open reading frame (ORF) 8, 7a, and 3b proteins [10]. Abs reactive to the membrane-distal S1 subunit of S protein, particularly against the N-terminal domain (NTD) and receptor-binding domain (RBD), are known to neutralize the virus [11,12,13,14].

While the role of neutralizing Abs against the membrane-distal S1 subunit is well characterized and exhibits potent neutralizing activity against the SARS-CoV-2 virus, the neutralization and antiviral activity of Abs directed against the relatively conserved membrane-proximal S2 subunit is not fully elucidated. Several studies have shown that convalescent individuals generated a strong serum IgG response against S2 that is associated with boosted S (OC43) IgG levels [15,16]. Moreover, infection also expanded the S2-reactive IgG memory B cell (MBC) populations and is shown to be associated with an increase in the S (OC43)-reactive IgG MBC levels. This suggests that the S2-specific response was recalled from the pre-existing population of MBCs [6].

A study looking at immunization with different forms of S antigens in an animal model showed that immunization with S2 did not generate strong neutralization Abs against SARS-CoV-2 pseudovirus compared to immunization with S1 or RBD subunits [17]. A recent report characterized a monoclonal Ab (mAb) isolated from mouse immunized with S proteins from MERS-CoV and SARS-CoV-2 that could recognize all five human β-coronaviruses; however, this pan-β-coronaviral S2-reactive mAb could only neutralize pseudotyped viruses expressing S protein from a subset of animal and human β-coronaviruses but not S from SARS-CoV-2 [18]. Collectively, this highlights that S2-reactive Abs have the potential to offer broad therapeutic applications by mediating broad anti-coronaviral protection, likely through the FcR-mediated mechanisms.

Although numerous studies have characterized immune responses against SARS-CoV-2 S protein and parts thereof (RBD, S1, NTD, and S2), the complete picture of the response is still an area of active investigation [19,20]. In particular, the nature of recall response against SARS-CoV-2 proteins is still poorly understood. Guided by previous observations that the strong IgG response in both serum and cellular MBC compartments against the S2 subunit was significantly correlated with the IgG response against full-length S (OC43), we would expect that broad S2-reactive MBC populations would be recalled to expand early in the acute phase. In addition to understanding the characteristics of recall response in the context of SARS-CoV-2 infection, it is imperative to understand the formation, expansion, breadth, and durability of specific MBC populations. 

The field has made substantial progress characterizing the immunological T and B memory following SARS-CoV-2 infection and vaccination [7,21,22,23]. In this study, we present a comprehensive analysis of the B cell response on a small cohort of individuals that we followed throughout the acute phase of infection until 3 months post symptom onset. Our analysis encompasses the serum Abs response, the plasmablast (PB) response, and the formation of MBC reactive to coronavirus antigens with the emphasis on the kinetics, magnitude, and breadth of specificities of the response. Additionally, we measured the chaotropic resistance of coronavirus-reactive IgG and antigen binding, which provides an insight on the avidity and by extension, the status of affinity maturation, on both serum and memory compartment.

## 2. Results

### 2.1. SARS-CoV-2 Infection Generates IgG against S and N Proteins

We analyzed samples that were collected from a small cohort (n = 8) of symptomatic non-hospitalized acutely infected individuals that we followed through five sampling time points (Table S1). Reactivity of circulating serum IgG was measured by ELISA with a panel of SARS-CoV-2 antigens that include full-length S ectodomain, receptor-binding domain (RBD), S2-only ectodomain subunit, and N proteins. S proteins from seasonal human α-coronavirus 229E and β-coronavirus OC43 were also included. Influenza H1 hemagglutinin (HA) was included as non-coronavirus control protein since most adults are routinely exposed to H1 either through vaccination or infection.

**Figure 1:**
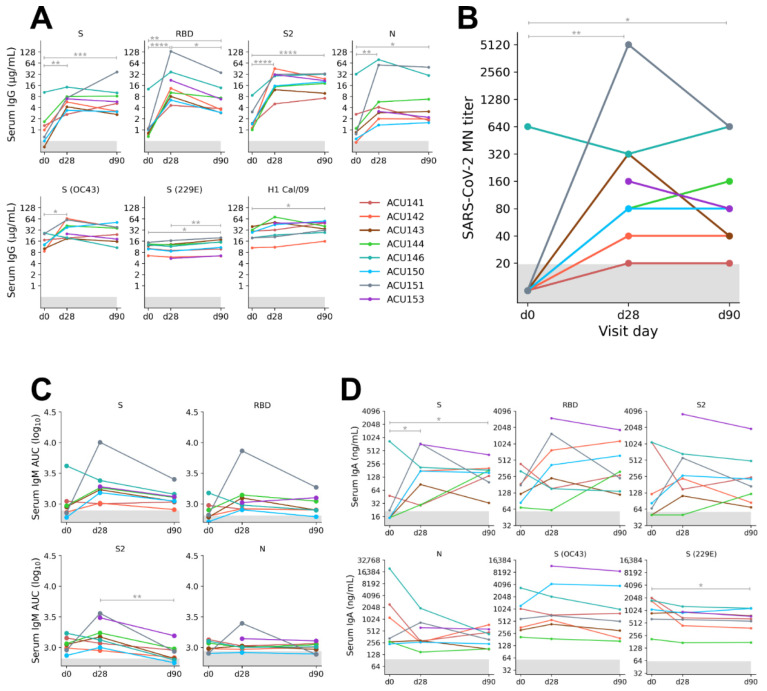
Serum Ig levels against SARS-CoV-2 and non-SARS-CoV-2 proteins in acutely infected cohort (n = 8), sampled on visit days 0, 28, and 90. (**A**,**C**,**D**) Serum IgG, IgM, and IgA levels were measured by ELISA and the assigned cutoff for positivity is shown by the horizontal gray bar. (**A**) Serum IgG concentrations measured by ELISA against the SARS-CoV-2 spike (S), receptor-binding domain (RBD), S2 subunit, nucleocapsid (N), S proteins from representative human seasonal α and β-coronaviruses OC43 and 229E, respectively. H1 Cal/09 protein was used as non-coronavirus control. (**B**) Levels of endpoint neutralization titer were measured by microneutralization (MN) assay with wild-type SARS-CoV-2 under BSL3 condition. (**C**) Levels of serum IgM measured by ELISA against SARS-CoV-2 S, RBD, S2, and N, and quantified by using area under the curve (AUC, log_10_-transformed). (**D**) Serum IgA concentrations measured by ELISA against the SARS-CoV-2 S, RBD, S2, N and S from seasonal coronaviruses OC43 and 229E. Significance (*, *p* < 0.05; **, *p* < 0.01; ***, *p* < 0.001; ****, *p* < 0.0001) between time points was determined by ANOVA modeled for linear mixed effects, followed by Tukey’s multiple comparisons test. Only significant comparisons are indicated on the plot.

Generally, all subjects generated a strong serum Ab response against all SARS-CoV-2 proteins tested (Figure 1A,C,D). The rise of IgG levels against SARS-CoV-2 antigens was accompanied by an increase in neutralization titer against wild-type SARS-CoV-2 virus (Figure 1B). There was also an increase in levels of antigen-specific IgM although the magnitude of the IgM response was small for most SARS-CoV-2 proteins (Figure 1C). The heterogeneity in the levels of antigen-specific IgA likely reflecting individual variations for the response against the infection, and likely influenced by the duration of presymptomatic infection (Figure 1D). Due to the nature of SARS-CoV-2 infection with a longer and variable presymptomatic incubation period ranging from 4–9 days [24], most subjects already had detectable levels of antigen-specific Ig reactive to SARS-CoV-2 proteins on visit day 0, albeit at a low level.

**Figure 2:**
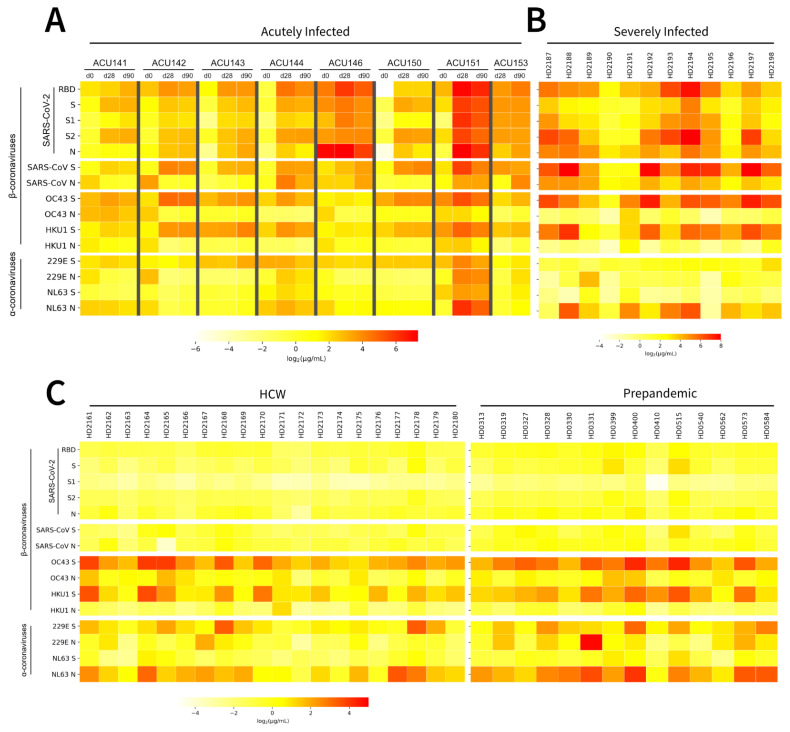
Breadth of serum IgG binding specificities reactive to S and N proteins from human coronaviruses SARS-CoV-2, SARS-CoV, OC43, HKU1, 229E, and NL63 measured by a multiplex-based assay. Antigen panel for S of SARS-CoV-2 includes full-length S and also RBD, S1, and S2 subunits. Data are presented as heatmap of log_2_-transformed concentration (μg/mL) values. (**A**) Sera from acutely infected cohort (n = 8) were sampled on visit days 0, 28, and 90. (**B**) Individuals previously hospitalized patients due to severe infection were sampled for a single time point as healthy donors (severely infected cohort, n = 12) between 8 to 20 weeks post symptom onset. (**C**) Healthcare workers (HCW, n = 20) were sampled during the pandemic while the prepandemic healthy individuals (n = 14) were sampled prior to the pandemic.

The significant 2.4-fold median increase (*p* = 0.015) in levels of S (OC43)-reactive serum IgG between days 0 and 28 is consistent with the notion of cross-reactive Ab response against conserved epitopes, likely targeting the S2 subunit on spikes of β-coronaviruses, whereas this pattern was not observed for the S (229E). To further interrogate the breadth of specificities of serum IgG, we tested sera against a range of coronavirus proteins (Table S2) by a multiplex-based assay. There was an increase in antigen-specific serum IgG levels reactive to SARS-CoV-2 proteins over time, consistent with our ELISA results (Figure 2A). As expected, there was an increase in levels of S (OC43) and S (HKU1)-reactive IgG throughout the sampling period, especially from visit days 0 to 28. There was also an increase in levels of S (SARS-CoV)-reactive IgG. This increase was expected, since SARS-CoV and SARS-CoV-2 share high sequence similarity among the human β-coronaviruses [25]. Thus, our analysis on the serum IgG response hinted at the recall response of pre-existing cross-reactive MBC compartment.

### 2.2. Unique Response Signature against N Protein

Surprisingly, there was an unexpected trend with the IgG reactivity profile against the N protein. We did not observe strong cross-reactive IgG response against N from seasonal β-coronaviruses. However, there was a strong IgG response against the N protein from α-coronavirus, notably against N (NL63) for subject ACU146 and ACU151 (Figure 2A). A recent study analyzed the serum Abs response against the N proteins from different HCoVs in different cohorts and concluded that reactivity to N proteins from human α- and β-coronavirus is influenced by the protein conformation and not strictly a function of sequence similarity, since N proteins from human α and β-coronaviruses possess low sequence similarity [26].

For a comparison with mild acutely infected individuals, we analyzed severely infected individuals (n = 12) that were hospitalized and received remdesivir treatment (Table S3). These convalescent individuals were sampled as healthy donors between 8 and 20 weeks post symptom onset. Most individuals showed robust serum IgG Abs response to SARS-CoV-2 S and N antigens, and to S proteins from β-coronaviruses. Similarly, there was also a significantly strong response reactive to N of NL63, but not to N of other seasonal coronaviruses (Figure 2B and Figure S1A). 

To compare the baseline of serum IgG composition in COVID-naïve populations, we re-analyzed two cohorts that were COVID-negative: healthcare workers (HCW) and pre-pandemic healthy donors [6]. As expected, there were minimal serum IgG reactivities against SARS-CoV-2 antigens and against SARS-CoV antigens. There were strong serum IgG reactivities against S proteins particularly S proteins of seasonal β-coronaviruses OC43 and HKU1 (Figure 2C). Notably, there was a strong serum IgG reactivity against N of NL63. By comparing the response against N of NL63, severely infected individuals showed boosted levels of serum IgG compared to the COVID-negative individuals (Figure S1B). Altogether, our multiplex analysis on the reactivity of serum IgG recognizing an array of coronavirus proteins allowed us to identify unique signature of the serum IgG response, notably the response against the spike from seasonal β-coronaviruses and specifically against the N of NL63.

### 2.3. Robust Increase in Serum IgG Avidity against Conserved Spike S2 Subunit

**Figure 3:**
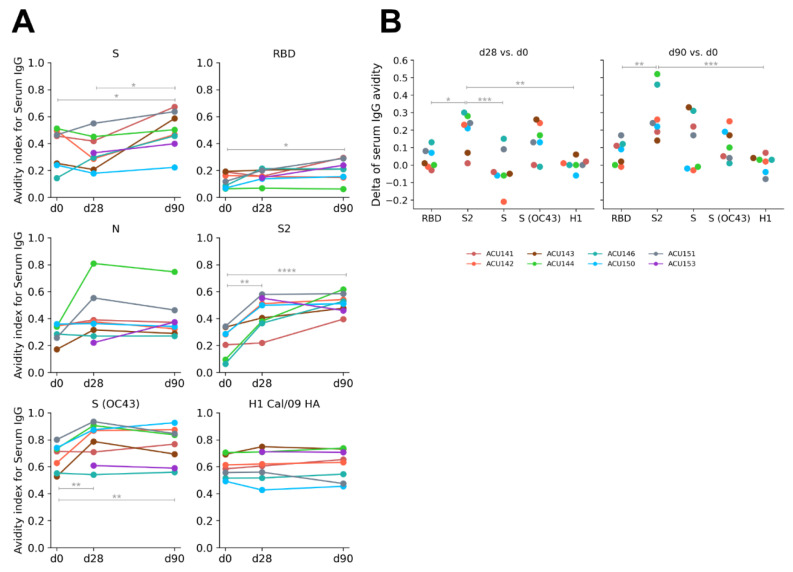
Chaotropic resistance of serum IgG (a proxy measure for avidity of antibody binding to protein antigen, shown as avidity index) measured by NaSCN displacement ELISA against SARS-CoV-2 S, RBD, N, S2 proteins, S (OC43), and H1 Cal/09 in acutely infected cohort (n = 8). (**A**) The kinetics of the avidity index measured for sera collected on visit days 0, 28, and 90. (**B**) Difference in the avidity index (visit days 28 vs. 0; 90 vs. 0) reactive to the indicated antigens. Significance (*, *p* < 0.05; **, *p* < 0.01; ***, *p* < 0.001; ****, *p* < 0.0001) was determined by ANOVA modeled for linear mixed effects, followed by Tukey’s multiple comparisons test. Only significant comparisons are indicated on the plot.

We then assessed the change in avidity of serum IgG against SARS-CoV-2 proteins, by using chaotrope resistance ELISA, for the acutely infected individuals. For this analysis, we included S (OC43) as well as H1 HA proteins. There were significant increases in the avidity of serum IgG reactive to the full-length S and RBD proteins (Figure 3A). The avidity of RBD-reactive serum IgG increased at a slower kinetics and a smaller magnitude between visit days 0 and 90 with 27.4% average increase (*p* = 0.016) compared to 34.7% average increase (*p* = 0.041) of the S-reactive serum IgG for the same period. 

As anticipated, there was a robust increase in avidity for S2-reactive IgG even between visit days 0 and 28 with 90% average increase (*p* = 0.0013). This was also accompanied by a similarly robust increase in avidity for S (OC43)-reactive serum IgG with 16.1% average increase (*p* = 0.003) for the same period. There was no significant increase in avidity for N- and H1-reactive serum IgG. By analyzing the difference in avidity either between visit days 28 or 90 to visit day 0, there was a clearer trend on the robust increase in avidity of S2- and S (OC43)-reactive serum IgG compared to other antigens that we tested (Figure 3B). Thus, the robust increase in avidity for S2 and S (OC43)-reactive serum IgG further supports the notion that these Abs were produced by pre-existing cross-reactive β-coronavirus memory pool and potential adaptation of the response to the S2 subunit.

### 2.4. Rapid Induction IgG Plasmablasts Recognizing Conserved Spike Subunit

At the early phase of infection, the Ab response to the infecting virus is produced by pre-existing cross-reactive MBC populations that were induced to differentiate into circulating antibody-secreting plasmablasts (PBs), therefore detectable in peripheral blood [27]. Our data on the increase in S2 and S (OC43)-reactive serum IgG levels and avidity profile provide strong evidence on the induction of broad coronavirus-reactive MBCs during the acute phase of infection. Thus, we sought to investigate the breadth of specificities of circulating PBs during the early phase of infection with the expectation that rapid activation of cross-reactive MBCs would lead to detectable circulating antigen-specific PBs in the peripheral blood.

To enumerate the frequency of antigen-specific IgG PBs, we performed enzyme-linked immunospot (ELISpot) assay on PBMCs sampled on visit days 0 and 3 and we tested their reactivities against SARS-CoV-2 proteins, along with S (OC43) and H1 HA proteins. In addition to enumerating the frequency of antigen-specific IgG PBs, we also assessed the reactivity of IgG Abs produced by PBs (polyclonal PB-derived Abs, PPAbs). As previously mentioned, due to the heterogeneity of the presymptomatic incubation period, infected individuals in our study cohort may have been infected for about 5–9 days before their first visit.

**Figure 4:**
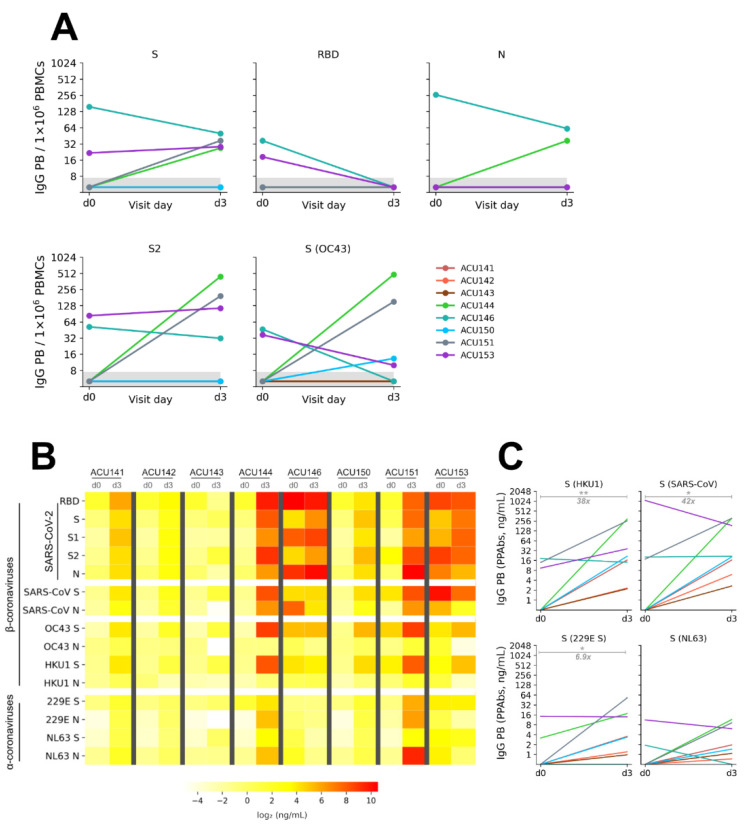
Coronavirus-specific IgG plasmablast (PB) response induced by SARS-CoV-2 infection in acutely infected cohort (n = 8). (**A**) Kinetics of the appearance of antigen-specific IgG PB in the peripheral blood for PBMCs sampled on visit days 0 and 3 for the indicated antigens measured by ELISpot. (**B**) Breadth of specificities of polyclonal Abs secreted by IgG PB (termed as PB-derived polyclonal Abs, PPAbs) against S and N proteins from human coronaviruses measured by a multiplex-based assay. (**C**) Levels of IgG PPAbs reactive against S proteins from HKU1, SARS-CoV, 229E, and NL63 showing the difference between visit days 0 and 3 measured by a multiplex-based assay, with fold change of median value on visit days 0 and 3 shown as a measure of effect size. Significance (*, *p* < 0.05; **, *p* < 0.01) was determined by paired *t* test.

There were coronavirus-reactive IgG PBs on both visit days 0 and 3, with generally higher frequency on visit day 3 for most subjects (Figure 4A). The frequencies of S2 and S (OC43)-reactive IgG PBs were higher on visit day 3 at 19.2-fold and 8.3-fold median increase, respectively, compared to visit day 0. Of note, the same four subjects that showed high frequency of IgG PBs reactive to S2 subunit demonstrated similarly high frequency of IgG PBs reactive to full-length S and S (OC43) at both sampling time points (Figure S2A). When assessing coronavirus-reactive IgG PBs as a fraction of total IgG PBs at both sampling time points, it was clear that S2 and S (OC43)-reactive IgG PBs demonstrated a larger response compared to other SARS-CoV-2 proteins that we tested (Figure S2B). This suggests that conserved epitopes present on the S2 subunit are the key targets of the B cell response during the early phase of SARS-CoV-2 infection.

We then assessed the breadth of specificities of IgG PPAbs by using the same multiplex-based assay (Figure 4B). We confirmed that the IgG PB response predominantly recognized SARS-CoV-2 proteins as the reactivity against H1 HA was below the detection limit (Figure S2C). As expected, there was an increase in levels of SARS-CoV-2-reactive IgG PPAbs from visit days 0 to 3. More importantly, the levels of β-coronavirus-reactive IgG PPAbs robustly increased. Similar to the increase in the frequency of S2 and S (OC43)-reactive IgG PBs that we measured by ELISpot (Figure 4A), there was a dramatic increase in the levels of S (HKU1)- and S (SARS-CoV)-reactive IgG PPAbs at 38-fold (*p* = 0.009) and 42-fold (*p* = 0.029), respectively, of median increase between visit days 0 and 3 (Figure 4C). We therefore conclude that conserved epitopes within the S2 subunit of β-coronavirus spike proteins are recognized by the pre-existing pool of broad coronavirus-reactive MBC populations, which then led to their induction into IgG PB during the acute phase of infection.

### 2.5. Rapid Increase in Formation of Coronavirus-Reactive MBCs Recognizing Conserved S2 Subunit

To assess the kinetics of the formation and the magnitude of response of coronavirus-reactive MBCs, we analyzed PBMCs sampled on visit days 0, 10, 28, and 90 from the acutely infected individuals, as well as PBMCs from individuals that were previously severely infected. We measured coronavirus-reactive IgG MBCs by in vitro stimulation to induce their differentiation into Ab-secreting cells (ASCs) [28], whereby we assessed the levels of MBC-derived ASCs (MASCs) by enzyme-linked immunosorbent spot (ELISpot) assay or MBC-derived polyclonal Abs (MPAbs) by the same multiplex-based assay. We previously validated the approach of using MPAbs to measure coronavirus-specific precursor IgG MBCs [6].

**Figure 5:**
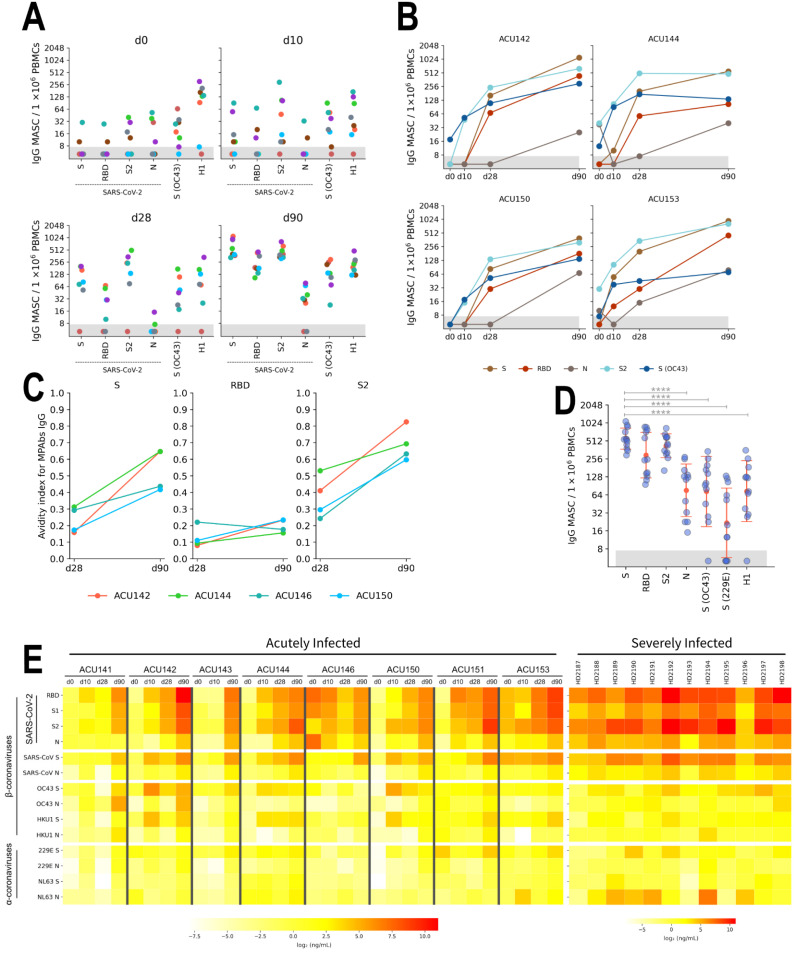
Analysis of IgG memory B cells (MBCs) reactive to SARS-CoV-2 proteins in acutely infected individuals (n = 8) (**A**,**B**,**C**,**E**) and healthy individuals who were previously hospitalized due to severe infection (severely infected cohort, n = 12) (**D**,**E**). Peripheral blood mononuclear cells (PBMCs) were sampled on visit days 0, 10, 28, and 90 for the acutely infected individuals. PBMCs for individuals from the severely infected cohort were sampled for a single time point between 8 to 20 weeks post symptom onset. PBMCs were stimulated in vitro to induce MBC differentiation into Ab-secreting cells, termed as MBC-derived Ab (IgG)-secreting cells (MASCs). (**A**) Formation and expansion of IgG MASCs reactive to SARS-CoV-2 S, RBD, S2, and N proteins, as well as reactive to S (OC43) and HA from H1 Cal/09 proteins for acutely infected subjects. (**B**) The kinetics of the formation of coronavirus-reactive IgG MBCs shown by the number of IgG MASCs for select subjects (ACU142, ACU144, ACU150, and ACU153) that exhibited high stimulation efficiency-based upon the frequency of total IgG MASCs. (**C**) Chaotropic resistance of post-stimulated MBC-derived polyclonal Abs (MPAbs) IgG (shown as avidity index) measured by NaSCN displacement ELISA against SARS-CoV-2 S, RBD, S2 and S (OC43) for select subjects, shown for visit days 28 and 90. Only subjects (n = 4) with sufficient MPAbs volume were tested. (**D**) IgG MASCs reactive to the same panel of coronavirus proteins with the additional S (229E) protein of the severely infected cohort. Range shows geometric mean ×/÷ geometric standard deviation. (**E**) Breadth of IgG MPAbs binding specificities reactive to S and N proteins from human coronaviruses measured by a multiplex-based assay for the acutely infected and severely infected cohorts as indicated. Data are presented as heatmap of log_2_-transformed concentration (μg/mL) values. Significance (****, *p* < 0.0001) was determined by ANOVA followed by Dunnett’s multiple comparisons test, comparing SARS-CoV-2 S against other proteins for panel D. Only significant comparisons are indicated on the plot.

We tested the reactivity of IgG MASCs against SARS-CoV-2 proteins, together with S (OC43) as well as H1 HA. On visit day 0, there were IgG MASC reactive to S (OC43), S2, and N at median frequency of 22.5, 8.75, and 6.25 IgG MASC per million PBMCs, respectively (Figure 5A).Throughout the acute infection phase, we observed strong correlation on the frequency of S2 and S (OC43)-reactive IgG MASCs (Figure S3), notably on visit days 10 (r*_p_* = 0.87, *p* = 0.005) and 28 (r*_p_* = 0.84, *p* = 0.019), suggesting that these populations of precursor MBCs share the same specificities. There was also a robust increase in S, RBD, and S2-reactive IgG MASCs on visit day 90 (Figure 5B). Analysis of the breadth of IgG response was performed by the same multiplex-based assay where there was a similar kinetics on the increase in IgG MPAbs levels against SARS-CoV-2 proteins, as well as against S proteins of the β-coronaviruses SARS-CoV, OC43, and HKU1 (Figure 5E, left panel).

The increase in the frequency of N-reactive MBCs, however, significantly lagged compared to S-reactive MBCs at 15-fold lower median frequency on visit day 90. This observation on the low magnitude of the formation of N-reactive MBCs is in line with previous studies analyzing the frequencies of post-stimulated SARS-CoV-2-reactive MBCs in convalescent individuals [6,7]. Additionally, within the memory compartment, there was an increase in avidity, as measured by chaotrope resistance ELISA, of the IgG Abs secreted by post-stimulated MBCs especially between visit days 28 and 90 for S, RBD, and S2-reactive IgG MPAbs (Figure 5C). This indicates adaptation of the MBC response to SARS-CoV-2 proteins and the on-going germinal center reaction from the infection.

We also assessed the formation of coronavirus-reactive MBCs in individuals from the severely infected cohort (Figure 5D). Similarly, they all demonstrated robust frequency of S, RBD, and S2-reactive MBCs and with heterogeneity in the frequency of N-reactive MBCs that is at 4.5-fold lower median frequency (*p* = 0.001). Similar to our finding for the acutely infected cohort, there was also a robust MBC response against all of the SARS-CoV-2 proteins as well, indicating that severe SARS-CoV-2 infection still leads to strong formation of immune memory, likely durable for a long period of time (Figure 5E, right panel). Altogether, our data suggests that SARS-CoV-2 infection, regardless of severity, leads to formation of SARS-CoV-2-reactive MBCs with some cross-reactivity against S proteins from other human β-coronaviruses.

## 3. Discussion

In this study, we sought to comprehensively characterize the cross-reactive MBC response in individuals that were infected by SARS-CoV-2. Our study on the cohort of acutely infected individuals that we tracked for 3 months allowed us to interrogate the kinetics of B cell response by assessing serum Abs, PBs, and MBCs. Additionally, we also assessed durability of coronavirus-reactive MBCs in healthy individuals that were previously severely infected. Here, we offer the following key findings: (i) the increase in the coronavirus-reactive IgG binding avidity in both serum and MBC compartments, (ii) the unique signature of the N-reactive response, (iii) the broad coronavirus-reactive IgG PB response during acute phase indicating the activation of pre-existing cross-reactive MBCs, and (iv) the rapid formation of S2-reactive IgG MBCs following infection.

The increase in S (OC43)-reactive serum IgG early during the acute phase infection indicates a cross-reactive serum Ab response, suggesting that response to SARS-CoV-2 might have recalled broad coronavirus-reactive pools to respond to the infection. In fact, several groups have discovered response to conserved cross-reactive epitopes on S proteins from β-coronaviruses [20,29]. Studies have described antiviral effects of the S2-reactive response demonstrating the benefits of the recall response [30,31,32], while others have shown S2-reactive Abs modulated the SARS-CoV-2 sterilizing response [33,34]. The robust response to S2 subunit may have been due to the competition of pre-existing cross-reactive MBCs with naïve B cells, reminiscent of cross-reactive response to head vs. stalk in influenza infection against novel HA subtypes [35,36,37]. Indeed, our observation on the PBs response at acute phase showed markedly strong induction of S2-reactive IgG PBs on visit days 0 and 3 compared to the SARS-CoV-2 RBD-reactive IgG PBs. This reflects the recall of broad coronavirus-reactive IgG MBCs early in the infection. Indeed, MAbs cloned from IgG PBs were highly mutated but modestly neutralized SARS-CoV-2 infection [19]. While it has been shown that SARS-CoV-2 infection induces strong IgA PB response accompanied with similarly robust IgA neutralization capacity [38], the role of S2-reactive response at the mucosal interface awaits further characterization.

We also observed a variable IgG PB response against the RBD early during infection, likely influenced by the heterogeneity in presymptomatic incubation period that led to the detection of RBD-reactive IgG PB at earlier sampling visits. Alternatively, it has been shown that human populations are poised to response to RBD based on multiple studies describing the public IGHV3-53 gene segment being overrepresented in convalescent individuals with the remarkable feature of requiring minimal mutation to achieve high affinity binding [39,40,41,42,43]. Our observation on the SARS-CoV-2-reactive MBCs also showed early formation of the RBD-reactive IgG MBCs, consistent with the notion that RBD-reactive B cells can emerge rapidly following exposure.

Several studies have reported that biased humoral N-reactive response could lead to poor prognosis [44,45]. In this study, we described a contrasting pattern between the serum and the MBC compartment reactive to SARS-CoV-2 N. IgM response to N exhibited lower magnitude, consistent with what have been previously reported [38], raising questions on the characteristics of N-reactive B cells that respond to infection. To our surprise, we observed high levels of N (NL63)-reactive IgG Abs in infected individuals as well as COVID-naïve individuals. However, less is known about the difference in immunogenicity of the response against N from other human coronaviruses and how pre-existing N-reactive Abs modulate response to infection. More importantly, we showed that the kinetics of the formation of N-reactive IgG MBCs significantly lagged compared to that that of S-reactive MBCs and subunits thereof. Thus, we believe that investigation into the formation of N-reactive MBC would be of an interest to fully elucidate generation of immune memory against SARS-CoV-2. 

Despite the decline in levels of serum Ab months post-exposure as the humoral response contracts back to homeostasis, generation of immune memory continues with improved qualities and can recognize variant viruses [46,47,48,49,50]. Here, we characterized robust formation of SARS-CoV-2-reactive IgG MBCs regardless of disease severity, especially the remarkable speed at which S2-reactive IgG MBCs were formed. We also showed that months after infection, Abs produced by post-stimulated MBCs possess high avidity binding to SARS-CoV-2 S, consistent with the on-going germinal center reaction months after recovery.

While we observed strong recall response reactive to the S2 subunit in the context of infection, the recall response in vaccinated individuals remains understudied. A recent study underscored that a two-dose mRNA vaccination regimen precludes strong recall response to other human β-coronaviruses [51]. We therefore hypothesized the difference in the response could have been attributed to design of the S immunogen: immunization with stabilized prefusion S conceals key S2 epitopes, therefore occluding recognition by pre-existing cross-reactive MBCs. On the other hand, the characteristics of these cross-reactive pre-existing MBCs are still relatively unexplored. A recent study characterized that mild infection generates SARS-CoV-2-reactive MBCs with robust phenotype [52], likely to be recalled upon re-exposure. The question as to whether pre-existing S2-reactive MBCs possess similarly robust phenotypes and are transcriptionally poised to respond to SARS-CoV-2 infection remains open.

## 4. Methods

### 4.1. Study Participants and Clinical Samples

All participants were recruited at the University of Rochester Medical Center in Rochester, New York, and provided written informed consent prior to inclusion in the studies. The studies were approved by the University of Rochester Human Research Subject Review Board (protocol 14-0101) and conducted in accordance with Good Clinical Practice. A cohort of 8 non-hospitalized PCR-confirmed SARS-CoV-2-infected individuals (median age, 44 years; interquartile range (IQR), 26 to 53 years) were enrolled in May 2020 (Table S1). Samples were collected on day 0 at disease presentation (visit 1), day 3 (visit 2), day 10 (visit 3), day 28 (visit 4), and day 90 (visit 5). Additionally, a cohort of previously hospitalized patients (n = 12; median age, 70 years; IQR, 56.5 to 80 years) with severe symptoms and received remdesivir treatment during hospitalization were enrolled into healthy donor study (protocol 14-0064) between 8 to 20 weeks after symptom onset (Table S3) to assess the durability antigen-specific MBC populations and their breadth of reactivities. A cohort of COVID-19-negative healthcare workers (n = 20) and prepandemic healthy donors (n = 14) were re-analyzed from Nguyen-Contant et al. 2020 [6].

### 4.2. Recombinant Proteins

RBD protein subunit from SARS-CoV-2 (isolate Wuhan-Hu-1) plasmid construct was kindly provided by Dr. Florian Krammer from Icahn School of Medicine at Mt. Sinai. Stabilized prefusion spike protein from SARS-CoV-2 (Wuhan-Hu-1) with 6 stabilizing proline mutations (HexaPro) plasmid construct was kindly provided by Dr. Jason McLellan from UT Austin (Addgene, Watertown MA, USA [53]). Both constructs were expressed in-house in Expi293F cell line (Gibco, Gaithersburg, MD, USA). Baculovirus-expressed S2 subdomain and HEK293-expressed N protein were obtained from SinoBiological (Chesterbrook, PA, USA) and RayBiotech (Peachtree Corners, PA, USA), respectively. Baculovirus-expressed S proteins from seasonal HCoVs OC43 (Chesterbrook, PA, USA) and 229E (Chesterbrook, PA, USA) were obtained from SinoBiological. In-house Expi293F-expressed hemagglutinin (HA) from egg-derived H1N1 A/California/7/2009 was used as noncoronavirus control antigen.

### 4.3. MBC Analysis

Levels of antigen-specific MBCs were measured as previously described [28]. Briefly, cryopreserved PBMC samples were thawed and incubated overnight in complete medium. The samples were then stimulated for 6 days at 1 × 10^6^ PBMCs/well in 24-well plates to induce MBC expansion and differentiation into MBC-derived ASCs (MASCs). The stimulation cocktail consisted of complete medium supplemented with 1 μg/mL R848 (St. Louis, MO, USA), 10 ng/mL IL-2 (Gibco, Gaithersburg, MD, USA), and 25 ng/mL IL-10 (STEMCELL Technologies, Vancouver, BC, Canada). After stimulation, cells were harvested and pelleted by centrifugation. The undiluted supernatant containing polyclonal Abs secreted by ASCs generated from stimulated MBC precursors (MPAbs) was collected and stored for analysis by ELISA or through a multiplex assay. Supernatants from unstimulated cultures were collected to control for Abs produced by pre-existing ASCs. Antigen-specific MASCs in the cell pellet were enumerated by ELISpot assay. For each antigen, 300 k stimulated PBMCs were analyzed by ELISpot assay and the limit of MASC detection was set at 7 MASCs per 10^6^ PBMCs. Antigen-specific IgG concentrations in MPAbs samples were also used as a measure of the relative sizes of reactive MBC populations

### 4.4. PB Analysis

Levels of antigen-specific PBs were measured as previously described [27]. Cryopreserved PBMCs were thawed into complete medium and counted. For each antigen, 400 k of PBMCs were directly seeded into ELISpot plates without prior B cell enrichment and incubated overnight. Separately, remaining PBMCs were cultured for 5 days in complete medium to collect PB-derived polyclonal Abs (PPAbs) and later analyzed by ELISA or through a multiplex assay.

### 4.5. ELISA

Concentration of antigen-specific IgG and IgA Abs were measured by ELISA and expressed as weight-based unit, as previously described [6]. Briefly, Nunc MaxiSorp 96-well plates (Thermo Scientific™, Waltham, MA, USA) were coated overnight with optimized concentrations of antigens. Samples were serially diluted in ELISA diluent buffer (1X PBS, 0.5% BSA, 0.05% Tween-20) and added to plates and incubated for 2 h at room temperature and then washed. Horseradish peroxidase (HRP)-conjugated anti-human IgG (Sigma, St. Louis, MO, USA) or anti-human IgA (Bethyl Labs, Montgomery, TX, USA) were added. After 2 h of incubation, TMB substrate (Thermo Scientific™, Waltham, MA, USA) was added. Color development was stopped with 2N H_2_SO_4_ and signal was acquired at 450 nm. The cutoff for positivity was set at 2× of the mean optical density (OD) value for negative wells. The performance of the assays is shown in supplemental Figure S4.

### 4.6. Coronavirus Multiplex Assay

Levels of binding IgG Abs from serum, MPAbs, and PPAbs were measured against a broad range of coronavirus proteins (Table S2) by multiplex assay, as recently described [54]. Sera was diluted to 1:1000 and Abs secreted by PBs and post-stimulated MBCs were diluted to 1:2, all with PBS. Samples were then incubated with bead panel mixture containing coupled HCoV S- and N-proteins for 2 h at room temperature, and then washed. Bead-bound Abs were detected using goat anti-human IgG (H + L) (Southern Biotech, Birmingham, AL, USA). Signal was acquired using Luminex MAGPIX™ Multiplex Reader. Concentration was assigned by nonlinear regression analysis using standard curves constructed by five-parameter logistic curve fitting

### 4.7. SARS-CoV-2 Microneutralization (MN) Assay

The MN assay to measure neutralization of infection by SARS-CoV-2-specific antibody was performed against SARS-CoV-2 isolate Hong Kong/VM20001061/2020 (BEI NR-52282) in BSL3 environment. Serum samples were first heat-inactivated at 56 °C for 30 min. Duplicates of heat-inactivated serum samples were serially diluted by 10-fold in virus diluent (DMEM, 5% FCS, 1% PSG, 20 mM HEPES) and incubated with 100 TCID_50_/mL of SARS-CoV-2 virus for 1 h at room temperature. The virus:sample mixtures (100 µL) were then added to confluent VeroE6/TMPRSS2 (a kind gift by Dr. Yoshihiro Kawaoka from University of Wisconsin-Madison) in 96-well flat-bottom plates containing 50 µL of post-infection medium (DMEM, 5% FCS, 1% PSG, 20 mM HEPES), then incubated at 37 °C, 5% CO_2_ for 48–72 h. Titer was determined by immunostaining using rabbit anti-SARS-CoV-2 N (GeneTex, Irvine, CA, USA) after fixation with 6% formaldehyde, followed by secondary Ab staining with goat anti-rabbit IgG HRP (Invitrogen, Waltham, MA, USA), and then developed with TMB substrate (Thermo Scientific™, Waltham, MA, USA).

### 4.8. NaSCN-Displacement ELISA

384-well plate wells (Thermo Scientific™, Waltham, MA, USA) were coated overnight with optimized concentration of antigens. Samples were serially diluted in ELISA diluent buffer and 15 μL of serially diluted samples were added to wells in duplicate (control vs. treated), followed by 2 h incubation at room temperature. 1.5 M NaSCN (Sigma, St. Louis, MO, USA, diluted in PBS) was added to treated wells at 20 μL/well, while ELISA diluent buffer was added to control wells. The samples were then incubated for 15 m at room temperature. Wells were washed 6x and dried. Bound Abs were detected using AP-conjugated mouse anti-human IgG MT78 (MabTech, Cincinnati, OH, USA), incubated for 2 h at room temperature. The reaction was developed using pNPP substrate (Thermo Scientific™, Waltham, MA, USA) and signal was acquired at 405 nm. Readout is expressed proportion of signal from NaSCN-treated wells over control wells of a serially diluted sample and denoted as avidity index [55].

### 4.9. Statistical Analyses

All statistical analyses were performed with GraphPad Prism v9 and R v4. All charts were generated using python visualization packages Matplotlib v3.3 and Seaborn v0.11.

## Data Availability

Data is available upon request.

## Supplementary Materials

The following supporting information can be downloaded at: https://www.mdpi.com/article/10.3390/pathogens11020186/s1. Table S1: Acute Infection Cohort; Table S2: Coronavirus Multiplex Antigen Panel; Table S3: Severely Infected Cohort; Figure S1: Characterization of serum IgG response reactive to nucleocapsid (N) proteins by multiplex-based assay. (S1A) Comparison of serum IgG response reactive to six N proteins of individuals that were severely infected (n = 12). (S1B) Comparison of the levels of N (NL63)-reactive serum IgG concentration between prepandemic, healthcare worker (HCW), and severely infected cohorts. Range shows geometric mean ×/÷ geometric standard deviation. Significance (*, *p* < 0.05; **, *p* < 0.01; ***, *p* < 0.001; ****, *p* < 0.0001) was determined by Kruskal-Wallis ANOVA followed by Dunn’s multiple comparisons test. The difference between levels is indicated as fold-change.; Figure S2: Coronavirus-specific IgG plasmablast (PB) response in acutely infected cohort. (S3A) Correlation analysis of the S2-reactive IgG PB against S (OC43) and full-length S. Correlation was tested by Pearson’s r on the log_2_-transformed IgG PB frequency per million PBMCs. Orange and red triangles represent IgG PB frequency readout on visit days 0 and 3, respectively. (S3B) Fraction proportion of antigen-specific IgG PB from the total IgG-producing cells for both visit days 0 and 3. (S3C) Assessing the reactivity of IgG PB (by ELISA on the PB-derived polyclonal Abs [PPAbs]) reactive to H1 control protein; Figure S3: Correlation between the frequency of SARS-CoV-2 S2- and S (OC43)-reactive IgG MASCs as enumerated by ELISpot for samples collected on visit days 0, 10, 28, and 90. Values shown are log_2_-transformed of the IgG MASC per million PBMCs. Correlation was tested by Pearson r; Figure S4: Validation of ELISA performance for protein constructs expressed in-house for (A) IgG and (B) IgA. 5-point serially-diluted sera (acutely infected cohort) were measured for reactivity against SARS-CoV-2 S (full-length), RBD, and H1 Cal/09. For IgA ELISA, COVID-negative controls (from healthcare worker cohort, indicated as gray lines) were included.
