# Supplementary material for: Formation and Expansion of Memory B Cells against Coronavirus in Acutely Infected COVID-19 Individuals
Source: Pathogens. 2022 Jan 29;11(2):186. doi: 10.3390/pathogens11020186 (PMC8876169; doi:10.3390/pathogens11020186)
Supplement: Supplementary file 1 [file pathogens-11-00186-s001.zip › pathogens-1543351-supplementary.pdf]

Table S1: Acute Infection Cohort

Participant ID	Birth Yr.	Age (Yr.)	Sex
ACU141	1995	25	M
ACU142	1966	53	F
ACU143	1966	54	M
ACU144	1990	29	F
ACU146	1966	53	F
ACU150	1972	47	F
ACU151	1979	40	M
ACU153	2000	20	F

All participants were PCR-positive for SARS-CoV-2 infection and presented with mild symptoms. All participants were enrolled in May 2020. “Age (Yr.)” column indicates age at enrolment.

Table S2: Coronavirus Multiplex Antigen Panel

Protein	Group	Virus
S	β-CoV	SARS-CoV-2
		SARS-CoV (2003)
		HCoV-OC43
		HCoV-HKU1
	α-CoV	HCoV-229E
		HCoV-NL63
N	β-CoV	SARS-CoV-2
		SARS-CoV (2003)
		HCoV-OC43
		HCoV-HKU1
	α-CoV	HCoV-229E
		HCoV-NL63
RBD*	β-CoV	SARS-CoV-2 (cat. #40592-V08H)
S1*		SARS-CoV-2 (cat. #40591-V08H)
S2*		SARS-CoV-2 (cat. #40590-V08H)

*Reagents purchased from SinoBiological.

Other reagents were expressed in-house using baculovirus expression system (Wang et al. 2021, Front. Immunol.).

Table S3: Severely Infected Cohort

Participant ID	Age (Yr.)	Sex
HD2187	76	M
HD2188	83	M
HD2189	20	F
HD2190	47	M
HD2191	81	F
HD2192	58	M
HD2193	73	F
HD2194	77	M
HD2195	82	F
HD2196	56	F
HD2197	67	F
HD2198	59	M

All participants were PCR-positive for SARS-CoV-2 infection and presented with severe symptoms (between March 2020 – June 2020) and required hospitalization, all received remdesivir treatment for a clinical study. All participants were enrolled in August 2020 as healthy donors. “Age (Yr.)” column indicates age at enrolment.

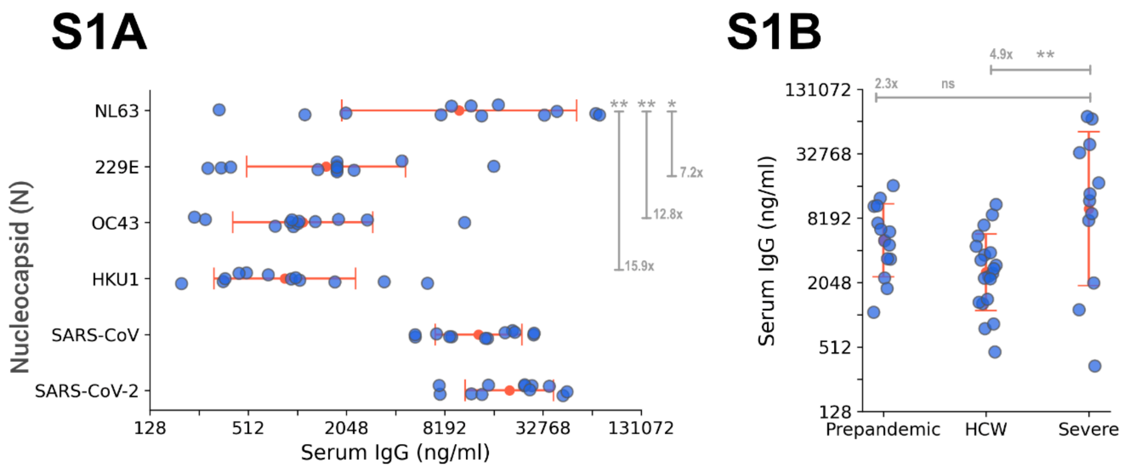

Supplemental Figure S1 | Characterization of serum IgG response reactive to nucleocapsid (N) proteins by multiplex-based assay. (**S1A**) Comparison of serum IgG response reactive to six N proteins of individuals that were severely infected (n=12). (**S1B**) Comparison of the levels of N (NL63)-reactive serum IgG concentration between prepandemic, healthcare worker (HCW), and severely infected cohorts. Range shows geometric mean ×/÷ geometric standard deviation. Significance (*, $P < 0.05$; **, $P < 0.01$; ***, $P < 0.001$; ****, $P < 0.0001$) was determined by Kruskal-Wallis ANOVA followed by Dunn’s multiple comparisons test. The difference between levels is indicated as fold-change.

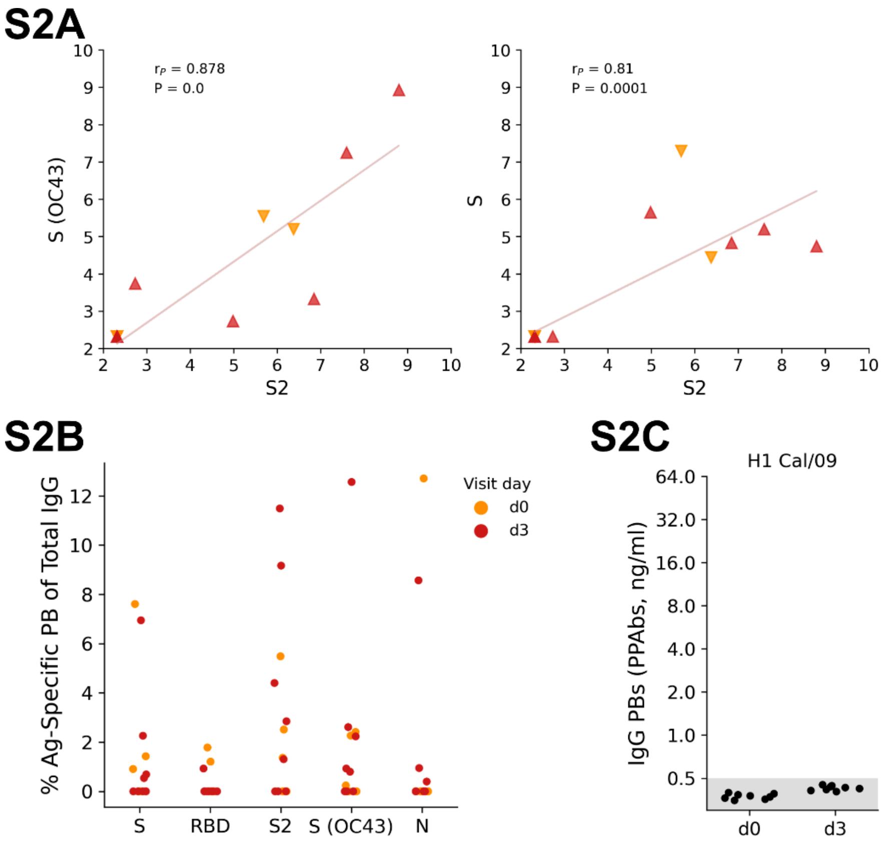

Supplemental Figure S2 | Coronavirus-specific IgG plasmablast (PB) response in acutely infected cohort. (**S3A**) Correlation analysis of the S2-reactive IgG PB against S (OC43) and full-length S. Correlation was tested by Pearson's r on the log_2-transformed IgG PB frequency per million PBMCs. Orange and red triangles represent IgG PB frequency readout on visit days 0 and 3, respectively. (**S3B**) Fraction proportion of antigen-specific IgG PB from the total IgG-producing cells for both visit days 0 and 3. (**S3C**) Assessing the reactivity of IgG PB (by ELISA on the PB-derived polyclonal Abs [PPAbs]) reactive to H1 control protein.

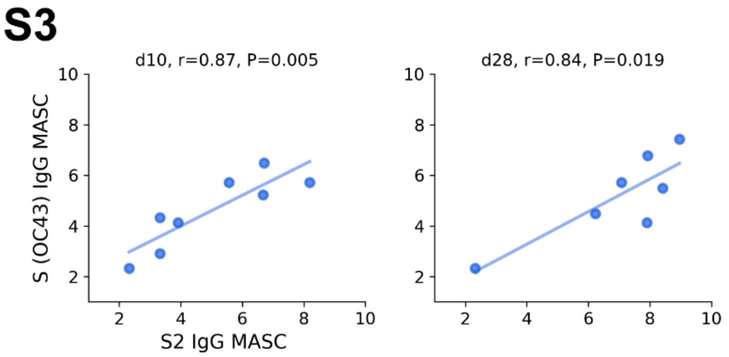

Supplemental Figure S3 | Correlation between the frequency of SARS-CoV-2 S2- and S (OC43)-reactive IgG MASCs as enumerated by ELISpot for samples collected on visit days 0, 10, 28, and 90. Values shown are log_2-transformed of the IgG MASC per million PBMCs. Correlation was tested by Pearson r.

S4A

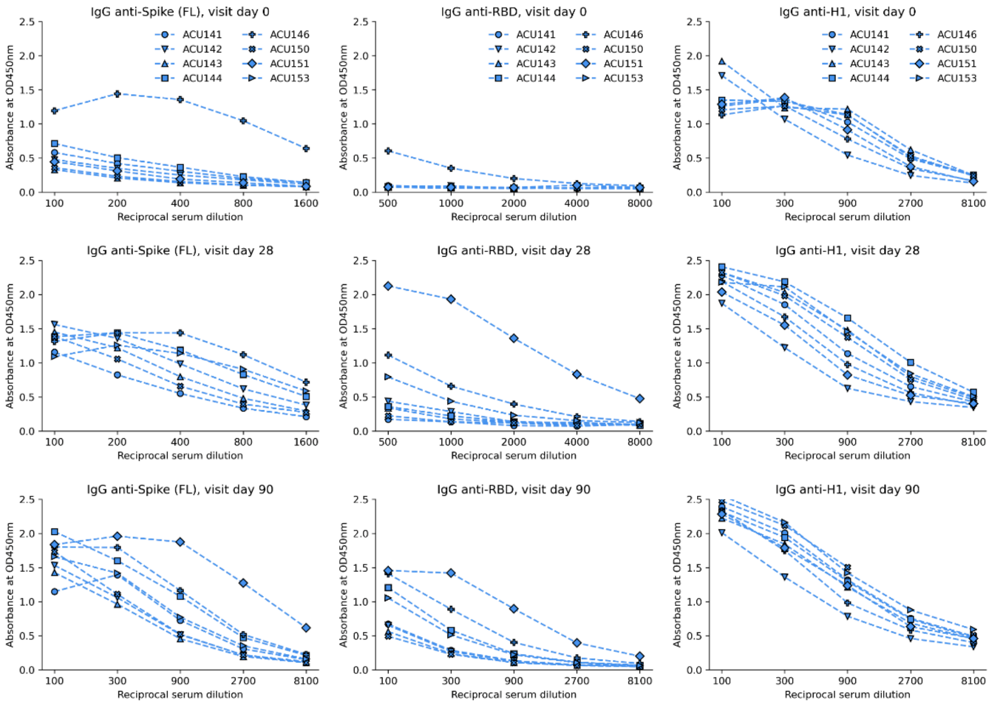

S4B

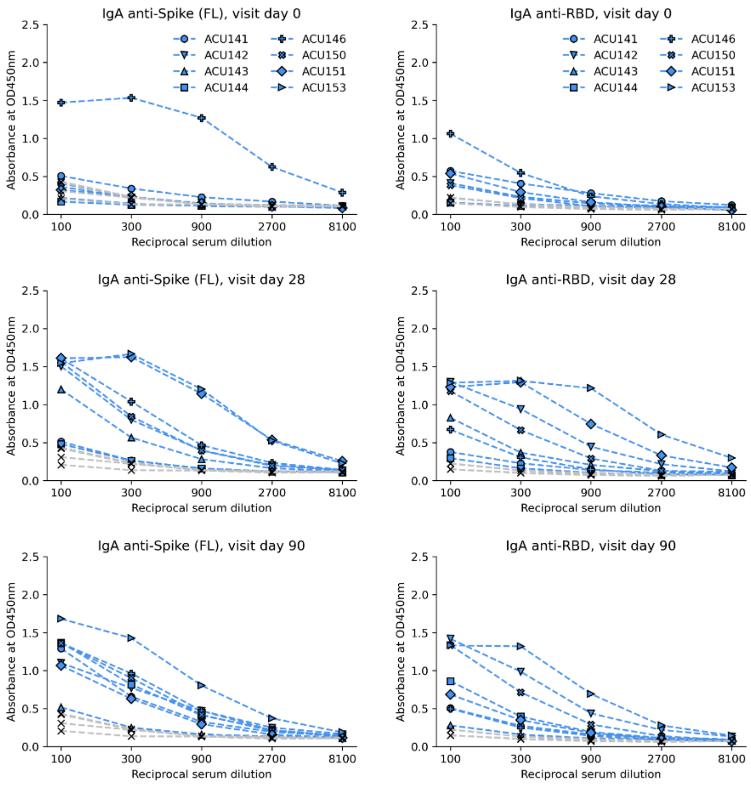

Supplemental Figure S4 | Validation of ELISA performance for protein constructs expressed in-house for (**A**) IgG and (**B**) IgA. 5-point serially-diluted sera (acutely infected cohort) were measured for reactivity against SARS-CoV-2 S (full-length), RBD, and H1 Cal/09. For IgA ELISA, COVID-negative controls (from healthcare worker cohort, indicated as gray lines) were included.
